# Association between Stress Urinary Incontinence and Depressive Symptoms after Birth: the Czech ELSPAC Study

**DOI:** 10.1038/s41598-020-62589-5

**Published:** 2020-04-10

**Authors:** Miluše Jurášková, Pavel Piler, Lubomír Kukla, Jan Švancara, Petra Daňsová, Lukáš Hruban, Vít Kandrnal, Hynek Pikhart

**Affiliations:** 1 Department of Rehabilitation Medicine, University Hospital Brno, Jihlavská 20, 602 00 Brno, Czech Republic; 2 RECETOX Centre, Faculty of Science, Masaryk University, Kamenice 5, 625 00 Brno, Czech Republic; 3 Institute of Biostatistics and Analyses, Faculty of Medicine, Masaryk University, Kamenice 3, 625 00 Brno, Czech Republic; 4 Institute for Research on Children, Youth and Family, Faculty of Social Studies, Masaryk University, Joštova 10, 602 00 Brno, Czech Republic; 5 Department of Epidemiology & Public Health, University College London, 1–19 Torrington Place, London, WC1E 6BT United Kingdom; 6 Department of Gynaecology and Obstetrics, University Hospital Brno, Jihlavská 20, 602 00 Brno, Czech Republic; 7 Department of Pharmacology, Faculty of Medicine, Masaryk University, Kamenice 753/5, 625 00 Brno, Czech Republic

**Keywords:** Epidemiology, Paediatric research

## Abstract

The study objectives were to (1) identify risk factors related to stress urinary incontinence (SUI) and postnatal depression (PD) after birth, and (2) investigate both possible directions of association between SUI and PD in population-based sample of Czech mothers. 3,701 nulliparous and multiparous women completed the self-reported questionnaires at 6 weeks and 6 months after birth and were included into the analyses of this prospective cohort study. Unadjusted and adjusted logistic regressions examined relationship between SUI a PD accounting for range of other risk factors. During the first 6 months after birth, 650 mothers (17.6%) developed SUI and 641 (17.3%) displayed signs of PD. The mode of delivery, parity and higher BMI were associated with SUI. The rate of PD symptoms was higher in mothers with positive history of prenatal depression, and in divorced or widowed mothers. Both conditions were associated with worse self-reported health, back pain and stop-smoker status. Initially, SUI at 6 weeks was slightly, but significantly associated with onset of PD at 6 months (OR 1.51, 95% CI 1.02–2.23) while PD at 6 weeks was not significantly related to new cases of SUI at 6 months (OR 1.48, 95% CI 0.91–2.39). After full adjustment these OR reduced to 1.41 and 1.38 (both non-significant), respectively. SUI and PD are common conditions in women postpartum that share some risk factors. Our study suggests that both directions of their relationship are possible although a larger study is needed to confirm our findings.

## Introduction

Urinary incontinence is a commonly experienced health problem in women after delivery. Its prevalence ranges between 18 and 34%^1^. Multiple studies showed that a vaginal delivery is the most significant postpartum urinary incontinence contributor^2^. Other suggested risk factors included increased maternal age, body mass index and parity^3^. In most cases urinary incontinence regresses spontaneously or after rehabilitation of the pelvic floor muscles. However, a substantial number of women with postpartum urinary incontinence are affected in the long term^4^. Postpartum urinary incontinence together with other health related problems such as tiredness, back pain, sexual problems, and relationship difficulties may contribute to the development of depressive symptoms^5^.

Transient mood disorders after birth are common but usually subside relatively quickly without any long term adverse effects^6^. Postpartum depression, on the other hand, is more severe mood disorder with a potential long term effect on mothers´ quality of life^7^. Its prevalence ranges from 5.2% to 13.0% in developed countries^8^ and can reach up to 20% in mothers from low and middle income countries^9^. In addition, some authors suggested that the rates reported in some studies were underestimated and would be higher if a standardized screening tool for depression in mothers after birth had been applied^10^. The main covariates for postnatal depression include personal and family history of depression and anxiety, severe medical comorbidities, lower socioeconomic status and excessive use of cigarettes^5,11^.

Three studies from the West confirmed association between urinary incontinence and postpartum depression^12–14^. In the cohort reported by Fritel^13^ the rate of newly diagnosed cases of depression was greater in subgroup of mothers with urinary incontinence. Melville *et al*. demonstrated that especially major depression could predict onset of urinary incontinence in a population of elderly women^15^. This hypothesis, however, has not been systematically studied in mothers postpartum. More detailed investigation of the direction and assessment of causality of the relationship between both conditions is important because these conditions are common, symptoms can be alleviated significantly and adequate treatment can improve not only quality of life of mothers but also development of the child^16,17^.

The objectives of our study were to determine rates of stress urinary incontinence and postnatal depression during the first 6 months postpartum, and to identify health and socioeconomic factors that could potentially contribute to the development of both stress urinary incontinence and postpartum depression. Additionally, we investigated both possible directions of association between stress urinary incontinence and postnatal depression in population-based sample of mothers from the Czech Republic, one of the Central European countries.

## Results

From the total of 3,701 women that were included in the analysis, 650 (17.6%) developed stress urinary incontinence during the first 6 months after birth; 641 (17.3%) displayed signs of postpartum depression during the same period (Table 1).

**Table 1:** Rate of stress urinary incontinence and depressive symptoms between birth and 6 months postpartum (n = 3,701 mothers).

Pathology	Number of cases	%
Stress urinary incontinence	650	17.6
Depressive symptoms	641	17.3

Among 3,701 mothers who completed the questionnaire related to stress urinary incontinence and depressive symptoms postpartum, 50% were pregnant for the first time and 40% were pregnant with the second child. The average age at the time of delivery was 25.3 years (standard deviation (SD) 5.0 years). The percentage of women with primary education was 37%. Only 20% of mothers had the university degree or an equivalent. With regards to the BMI before the pregnancy, 81% of mothers had BMI < 25 kg/m^2^, 11% of mothers were overweight and 3% obese. Prenatal depressive symptoms were reported by 480 (13%) mothers. The children were delivered mainly vaginally 90%, only 281 (8%) were delivered by means of the caesarean section (Table 2).

**Table 2:** Characteristics of the mothers and unadjusted odds of the stress urinary incontinence and depressive symptoms between birth and 6 months postpartum (n = 3,701 mothers).

Characteristics	N	%	Cases (%)	OR (95% CI) for urinary incontinence between birth and 6 months	p	Cases (%)	OR (95% CI) for depressive symptoms between birth and 6 months	p
**Maternal age in years**								
<19	419	11.3	13.4	1		19.1	1	
20–24	1,480	40.0	14.8	1.13 (0.82–1.54)		15.6	0.78 (0.59–1.04)	
25–30	1,229	33.2	18.7	1.49 (1.10–2.05)	*	17.7	0.91 (0.68–1.21)	
>30	573	15.5	25.3	2.20 (1.57–3.08)	***	19.7	1.04 (0.75–1.43)	
**Maternal education**								
Primary	1,371	37.0	14.9	1		17.8	1	
Secondary	1,619	43.7	18.0	1.26 (1.04–1.53)	*	16.3	0.90 (0.74–1.09)	
University	690	18.6	21.3	1.55 (1.22–1.96)	***	19.0	1.08 (0.86–1.37)	
Missing	21	0.6	33.3	—		9.5	—	
**Marital status**								
Married	3,257	88.0	17.6	1		16.9	1	
Divorced/widowed	106	2.9	20.8	1.23 (0.76–1.98)		26.4	1.76 (1.14–2.75)	*
Single	297	8.0	15.8	0.88 (0.64–1.22)		20.2	1.24 (0.93–1.68)	
Missing	41	1.1	22.0	—		7.3	—	
**Parity**								
0	1,857	50.2	15.1	1		16.8	1	
1	1,467	39.6	19.1	1.33 (1.11–1.59)	**	17.3	1.04 (0.87–1.25)	
2	292	7.9	21.9	1.58 (1.17–2.14)	**	19.5	1.21 (0.88–1.65)	
3+	85	2.3	30.6	2.48 (1.54–4.00)	***	22.4	1.43 (0.85–2.42)	
**Smoking status**								
Non-smoker	2,140	57.8	16.2	1		15.7	1	
Stop-smoker	1,246	33.7	19.7	1.27 (1.06–1.52)	**	19.3	1.29 (1.07–1.54)	**
Smoker	249	6.7	18.9	1.20 (0.85–1.69)		23.3	1.64 (1.19–2.24)	**
Missing	66	1.8	15.2	—		12.1	—	
**Pre-pregnancy BMI** **(BMI, kg/m**^**2**^**)**								
<18.4	286	7.7	11.5	1		17.8	1	
18.5–24.9	2,717	73.4	17.4	1.62 (1.11–2.35)	*	17.5	0.97 (0.71–1.34)	
25.0–29.9	413	11.2	21.6	2.11 (1.37–3.24)	**	17.4	0.97 (0.66–1.54)	
>30	106	2.9	27.4	2.89 (1.65–5.06)	***	20.8	1.21 (0.69–2.11)	
Missing	179	4.8	14.5	—		12.3	—	
**Back pain**								
During pregnancy	771	20.8	21.9	1.52 (1.24–1.87)	***	22.4	1.73 (1.40–2.12)	***
Only in past	531	14.4	19.8	1.34 (1.05–1.70)	**	21.3	1.61 (1.27–2.05)	***
No	2,222	60.0	15.6	1		14.4	1	
Missing	177	4.8	17.0	—		20.3	—	
**Pre-pregnancy self-reported health**								
Excellent	897	24.2	15.2	1		11.6	1	
Good	2,429	65.6	17.9	1.22 (0.99–1.51)	*	18.0	1.67 (1.33–2.10)	***
Poor	345	9.3	21.5	1.53 (1.11–2.09)	**	28.4	3.03 (2.22–4.13)	***
Missing	30	0.8	16.7	—		10.0	—	
**Prenatal depressive symptoms**								
Yes	480	83.4	21.7	1.34 (1.06–1.69)	*	44.0	5.18 (4.21–6.38)	***
No	3,087	13.0	17.1	1		13.2	1	
Missing	134	3.6	12.7	—		17.9	—	
**Mother wetting in later childhood (after age 5)**								
Yes	179	4.8	24.6	1.59 (1.12–2.26)	*	16.9	1.28 (0.88–1.86)	
No	3,373	91.1	17.0	1		20.7	1	
Missing	149	4.0	21.5	—		22.2	—	
**Depression in mother family history**								
Yes	610	16.5	21.6	1.45 (1.17–1.80)	**	26.1	1.96 (1.59–2.42)	***
No	2,707	73.1	16.0	1		15.2	1	
Missing	384	10.4	22.1	—		18.2	—	
**Birth weight**								
<2500	185	5.0	10.8	0.56 (0.35–0.90)	*	13.5	0.73 (0.48–1.13)	
2501–3900	3,118	84.3	17.8	1		17.6	1	
>3900	315	8.5	19.1	1.09 (0.81–1.46)		14.6	0.80 (0.58–1.11)	
Missing	83	2.2	19.3	—		26.5	—	
**Mode of delivery**								
Caesarean – induction	143	3.9	7.0	1		21.0	1	
Caesarean – acute	138	3.7	8.0	1.15 (0.47–2.81)		22.5	1.09 (0.62–1.92)	
Vaginal – spontaneous	3,276	88.5	18.4	3.00 (1.57–5.57)	**	16.7	0.75 (0.50–1.14)	
Vaginal – instrumental	67	1.8	17.9	2.90 (1.18–7.11)	*	17.9	0.82 (0.39–1.73)	
Missing	77	2.1	16.9	—		17.3	—	

*p < 0.05, **p < 0.01, ***p < 0.001.

The bivariate analysis identified factors associated with stress urinary incontinence during the first 6 months after birth. The most significant was the mode of delivery, parity and higher pre-pregnancy BMI (Table 2). The rate of postpartum depression was considerably higher in mothers with prenatal history of depression and mothers who were divorced or widowed. Both conditions were also associated with worse self-reported health, back pain and with stop-smoker status.

In unadjusted analysis, the stress urinary incontinence at 6 weeks postpartum was slightly, but significantly associated with onset of postpartum depression at 6 months after birth (OR 1.51, 95% CI 1.02–2.23) (Table 3). Presence of the depressive symptoms at 6 weeks postpartum increased the odds of urinary incontinence at 6 months after birth (OR 1.48, 95% CI 0.91–2.39) although this association was not statistically significant (Table 4). Slightly reduced magnitude of the effects was observed in the multivariable analysis although the results were not statistically significant for either of the directions.

**Table 3:** The association of stress urinary incontinence at 6 weeks with onset of depressive symptoms at 6 months after birth.

Onset of depressive symptoms at 6 months	N	% cases	Unadjusted OR (95% CI)	Adjusted OR (95% CI)
Stress urinary incontinence at 6 weeks	2,814	6.8	—	—
No	2,462	6.4	1	1
Yes	352	9.4	**1.51 (1.02**–**2.23)**	1.41 (0.93–2.15)

Mothers with depressive symptoms at 6 weeks after pregnancy were excluded. Adjusted for maternal age in years, maternal education, marital status, parity, smoking status, pre-pregnancy BMI, back pain, pre-pregnancy self-reported health, prenatal depressive symptoms, mother wetting in later childhood, depression in mother family history, birth weight, mode of delivery.

**Table 4:** The association of depressive symptoms at 6 weeks with onset of stress urinary incontinence at 6 months after birth.

Onset of stress urinary incontinence at 6 months	N	% cases	Unadjusted OR (95% CI)	Adjusted OR (95% CI)
Depressive symptoms at 6 weeks	2,785	5.0	—	—
No	2,482	4.8	1	1
Yes	303	6.9	1.48 (0.91–2.39)	1.38 (0.84–2.31)

Mothers with stress urinary incontinence at 6 weeks after pregnancy were excluded. Adjusted for maternal age in years, maternal education, marital status, parity, smoking status, pre-pregnancy BMI, back pain, pre-pregnancy self-reported health, prenatal depressive symptoms, mother wetting in later childhood, depression in mother family history, birth weight, mode of delivery.

## Discussion

In our longitudinal study, women with stress urinary incontinence at 6 weeks after birth had 1.5 higher risk of postpartum depression development at 6 months. Similar trends were observed in French prospective cohort study following 1,226 mothers postpartum reported by Fritel *et al*., where the risk of depression (EPDS ≥ 10) also increased 1.5-times in women with stress and urge urinary incontinence^13^. The association between these two conditions in mothers after delivery were also demonstrated by Brown and Hullfish in Australia and U.S. prospective studies^12,18^, as well as a retrospective study by Swenson who identified urinary incontinence during and after pregnancy as a risk factor for postpartum depression symptoms in the sample of U.S. mothers (adjusted OR 3.8)^14^.

There are several pathways through which postpartum depression can develop. Involuntary leakage of urine can be bothersome, lead to anxiety, and subsequently contribute to social isolation and depression development^19,20^. Dysregulation of serotonin after pregnancy due to decrease of estrogen levels is also a plausible explanation for both depression^21^ as well as incontinence development^22^. Increased activity of hypothalamic-pituitary axis and the sympathetic nervous system exhibit increased activity in many depressed individuals^23^ resulting in increased release of cortisol and catechol amines. In the longer term, this may lead to mood disruption as well as to physiologic changes of the bladder contributing to involuntary urine loss especially in the terrain of pelvic muscle and nerves damage.

The mechanism mentioned above could explain our results also suggesting the direction of causality from depressive symptoms to urinary incontinence. Mothers that suffered from postpartum depression at 6 weeks after birth were 38% more likely to develop urinary incontinence at 6 months than mothers who were not depressed. Even though our results were not statistically significant, they are in accordance with results from different population of women followed by the Health and Retirement Study reported by Melville^15^.

Our results showed that the most significant risk factors of postpartum stress urinary incontinence include vaginal birth, multi-parity, mothers´ age and increased body mass index before pregnancy. Relatively low rate of cesarean section births in our data (although virtually the same as nationally reported by Czech Statistical Office in 1991) might be potential limitation. Some earlier papers show that planned Caesarean section can reduce the rate of urinary incontinence but can be a cause of maternal complications including bladder injuries^24^. The low rate of Caesarean section may reduce the importance of this confounder but, at the same time, reduce the use of results in populations with higher rate of Caesarean section^25^. Similar results reported other studies^3,26^ but none of them described mutual risk factors for postpartum urinary incontinence and depression. The mutual risk factors for both conditions, according to our study, are poor self-reported health, back pain together with stop-smoker status. Based on this finding a group of mothers at risk could be defined and screened for both depression as well as postpartum urinary incontinence.

The rate of postpartum stress urinary incontinence observed in ELSPAC (17.6%) during the first six months after birth was in accordance with prevalence of postpartum stress urinary incontinence (21%) at five months introduced in the Altman study^27^. On the other hand the newest systematic review by Thom reported higher values (33%) of the pooled prevalence of any postpartum incontinence during the first 3 months after delivery probably due to methodological differences, namely the definition of incontinence and the time point when the incontinence was assessed^1^. In numerous studies distinction between stress and urge incontinence is unclear while we focused only on stress incontinence^12^.

The rate of postnatal depression (17.3%) was slightly higher than meta-analysis of a large number of studies^28^. However; as already highlighted some authors believe that the rates of postpartum depression are underestimated and would increase considerably if the standard screening was applied on the whole population^10^.

### Strengths and limitations

Our prospective study followed high number of mothers (3,701) from the region where the similar studies had not been conducted before. Standard validated self-reported scale (EPDS) was used at two different time points during the first six months postpartum to determine postnatal depression; standardized approach was also used to determine UI. Robust data about wide range of potential covariates were collected. The response rate (70%) as well as the rates of postnatal stress urinary incontinence and postnatal depression were comparable with other studies addressing similar outcomes. While it is possible that having approximately 30% of non-respondents could affect our findings we think these results should not be affected more than results of other studies on this topic.

One of the potential limitations of the study was that we screened for signs of stress urinary incontinence only up to 6 months after birth and therefore we could not assess long term development of this outcome. In addition, post hoc power analysis showed that to confirm the direction of relationship from SUI to PD with 95% confidence we would require total of 9360 mothers (respectively 1040 mothers with confirmed PD and 8320 without confirmed PD). While we understand that the study is not sufficiently powered, we feel that it is important to publish results from the Central/Eastern European region to reduce the publication bias regarding this topic. Relatively low analytical power also prevented us from performing detailed analysis of urinary incontinence cases at 6 months after birth. The fact that we based our study on the data from the 1990’s might also be perceived as another limitation. However, we feel that the mechanisms of postpartum urinary incontinence and depressive symptoms remain the same and our findings are applicable to the current population.

The main findings of our study contributed to the body of previous evidence describing relations between postnatal urinary incontinence and depressive symptoms. They confirmed relatively high rates in mothers during the first 6 months after birth. Despite the fact that regular screening for urinary incontinence and postpartum depression would increase time required for preventive procedures in mothers after delivery, we believe that it is worth considering because both conditions are common, amenable to specific interventions, their symptoms are recognizable without a special technology, and can be done by any trained healthcare professional.

## Methods

### Study population

Data for this analysis were derived from initial waves of the Czech part of the European Longitudinal Study of Pregnancy and Childhood (ELSPAC-CZ). From 7,895 eligible women (all pregnancies in selected geographical region within target period) the ELSPAC-CZ study recruited 5,151 mothers from Brno and Znojmo regions of former Czechoslovakia (now the Czech Republic) who expected to deliver a baby between 1st March 1991 and 30th June 1992. The study sample of 3,701 nulliparous as well as multiparous women completed self-reported questionnaires during pregnancy (predominantly week 20 +/− 2 weeks), at 6 weeks and 6 months after birth. An earlier publication describing the cohort profile shows that those included in the ELSPAC-CZ study do not differ substantially from the whole target population in terms of available demographic characteristics^29^. The questionnaires included the Edinburgh Postnatal Depression Scale (EPDS), specific questions to confirm stress urinary incontinence symptoms, a socio-economic status questionnaire, and a general health questionnaire. Some data used in this study were retrieved from the maternity hospitals database and health records collected by the study investigators. Informed consents were obtained from all study participants during the each wave of the data collection. An ethics committee approval for all aspects of data collection as well as for the secondary analysis was obtained from the ELSPAC ethics committee. This project has been approved by ELSPAC ethics committee under reference number 2017–039-Stan. Additional details about the study have been published earlier^29^.

### Data collection

#### Postpartum depression

Depressive symptoms of the ELSPAC mothers were assessed at 6 weeks and 6 months after birth using the Edinburgh Postnatal Depression Scale (EPDS). The EPDS is a standardized validated questionnaire for screening of prenatal and postnatal depressive symptoms repeatedly used by range of population studies^30^ including the “sister” ALSPAC study^31^. The EPDS is a 10-item questionnaire with responses to each item ranging from 0 to 3^32^. The overall score is constructed as simple sum, and ranges between 0–30. As in the original paper evaluating the Czech ELSPAC data related to postnatal depression^33^, we used a cut-off 10 in combination with a positive response to the question related to the depressive mood (“felt miserable and sad quite often”) for positive definition of postnatal depression, and we used this binary variable as a study outcome.

#### Postpartum stress urinary incontinence

Postpartum stress urinary incontinence was determined by a positive answer to the question in a self-reported questionnaire at 6 weeks and 6 months after birth (“Do you experience urinary leakage related to physical activity, coughing and/or sneezing after birth?”). The question is in line with the 2003 International Continence Society (ICS) definition of stress urinary incontinence^34^ and its validity and reliability was demonstrated by Rohr *et al*.^35^. Mothers were regarded as having postnatal stress urinary incontinence if they confirmed a urinary leakage at 6 weeks and/or at 6 months after birth, and did not report having urinary incontinence during pregnancy.

### Covariates used in the analysis

Information on potential covariates (potential confounders and mediators selected to reflect pathophysiological mechanism and based on review of current literature) was collected from the self-reported questionnaires and medical records from the maternity hospitals. Covariates included age of mother, education, marital status, parity, smoking habits, pre-pregnancy body mass index (BMI), information about back pain, self-reported health, prenatal symptoms, wetting in mother’s childhood after age 5 years, prenatal depressive symptoms, depression in mother’s family history, birth weight, or mode of delivery.

### Statistical analysis

Data were analyzed using the STATA software version 14 (Stata Corp, Texas, and U.S.). First, we described rates of stress urinary incontinence and postpartum depression between the birth and 6 months postpartum. For this description we defined a group of mothers who had at least one record of stress urinary incontinence and/or confirmed postpartum depression at 6 weeks and 6 months after delivery. Second, frequency distributions were used to describe all study variables for this group of mothers. Subsequently, we performed bivariate analysis to investigate association between each potential covariate and onset of stress urinary incontinence/depressive symptoms during the first 6 months after delivery. Third, we performed two unadjusted logistic regressions to examine both directions of the relationship between stress urinary incontinence and postpartum depression after delivery. In the first analysis, stress urinary incontinence at 6 weeks after birth was used to predict onset of postpartum depression at 6 months. Mothers with postpartum depression at 6 weeks were excluded from this analysis. In the second analysis, postpartum depression at 6 weeks after birth was used to predict onset of urinary incontinence at 6 months. Mothers with urinary incontinence at 6 weeks were excluded from this analysis. As a final step, we constructed adjusted logistic regression models including all potential risk factors and covariates.
